# Nurse-work instability and incidence of sick leave – results of a prospective study of nurses aged over 40

**DOI:** 10.1186/s12995-018-0212-y

**Published:** 2018-10-05

**Authors:** Melanie Klein, Stefanie Wobbe-Ribinski, Anika Buchholz, Albert Nienhaus, Anja Schablon

**Affiliations:** 1 Centre of Excellence for Epidemiology and Health Care Research for Health Care Workers (CVcare), University Medical Center Hamburg-Eppendorf (UKE), Martinistr. 41a, 20521 Hamburg, Germany; 2 DAK-Gesundheit (Health Insurance Fund, Board Manager for Health Care Research, Nagelsweg 27-31, 20097 Hamburg, Germany; 3 Institute of Medical Biometry and Epidemiology (IMBE), University Medical Center Hamburg-Eppendorf (UKE), Martinistr. 52, 20246 Hamburg, Germany; 4 Department of Occupational Health Research, German Social Accident Insurance Institution for the Health and Welfare Services, Pappelallee 33-37, 22089 Hamburg, Germany

**Keywords:** Nurse-work instability scale, Nurses, Long-term sick leave, Secondary data of a health insurer

## Abstract

**Background:**

The Nurse Work Instability Scale (Nurse-WIS) is an occupation-specific instrument that ascertains “work instability,” the interval before restricted work ability or prolonged sick leave occurs. The objective of the study was to assess if nurses with a high risk baseline-score in the Nurse-WIS take longer periods of sick leave due to musculoskeletal diseases and/or psychological impairments than other nurses.

**Methods:**

A total of 4500 nurses randomly selected from one of the largest health insurance funds in Germany (DAK-Gesundheit) were invited by letter to participate in the study. The participants answered a questionnaire at baseline and gave consent to a transfer of data concerning sick leave during the twelve months following completion of the questionnaire from the health insurance to the study centre. Sensitivity, specificity and positive and negative predictive values (PPV and NPV) for long-term sick leave were calculated. In order to analyze the association between the Nurse-WIS and sick leave during follow-up, a multiple ordinal logistic model (proportional odds model) was applied.

**Results:**

A total of 1592 nurses took part in the study (response 35.6%). No loss of follow-up occurred. The number of nurses with a high score (20–28 points) in the Nurse-WIS was 628 (39.4%), and 639 (40.1%) had taken sick leave due to musculoskeletal diseases or psychological impairment during the follow-up period. The odds ratio for sick leave in nurses with a high Nurse-WIS score was 3.42 (95%CI 2.54–4.60). Sensitivity for long-term sick leave (< 42 days) was 64.1%, specificity 63.4%, PPV 17.0% and NPP 93.8%.

**Conclusion:**

The German version of the Nurse-WIS predicts long-term sick leave, but the PPV is rather low. Combining questionnaire data with secondary data from a health insurer was feasible. Therefore further studies employing this combination of data are advisable.

## Background

Demographic transition will lead to an increase in the demand for nurses in many countries [1–3]. However, nurses frequently suffer from musculoskeletal diseases [4–10], psychological impairments, burnout, or poor general health [11–14]. According to the Health Report of one of the largest statutory health insurance funds in Germany, the DAK-Gesundheit, nurses more often take sick leave, and for longer periods than other insured groups [15]. Diseases of the musculoskeletal system and psychological impairments are the most frequent causes of long-term sick leave. Moreover, long-term sick leave is often an intermediate stage on the way to early retirement [16], which is frequent among nurses [17]. Therefore it is important to maintain nurses’ work ability. In order to achieve this goal, it appears sensible to offer interventions that allow nurses to remain healthy and motivated in their profession until retirement age. The most effective approach toward achieving this goal is to use multimodal interventions [18] or interventions that include persons with the initial symptoms of musculoskeletal disease [19–22]. However, no effective screening instrument has been available for early recognition of nurses at risk. The Nurse- Work Instability Scale (Nurse-WIS) is a questionnaire that seems to fulfill this requirement [23]. Work instability is the interval before restricted work ability when the subject has increasing difficulty in performing his or her duties at work, and can be ascertained with this occupation-specific instrument for nurses. Interventions during this interval can prevent impending loss of work ability. Thus early identification of work instability is the key to preventing the subject’s situation from deteriorating, with the resulting loss in work ability [24–26]. The development and validation of the German version of the scale, were performed using a cohort of geriatric care workers [27, 28]. The study showed that the German version of the scale is an easy, reliable and valid instrument with moderate prognostic ability to ascertain impending sick leave. The questionnaire has not yet been validated for nurses. Therefore one goal of the study presented here was to assess the performance of the Nurse-WIS in a cohort of nurses. In addition, the scale was updated because it is desirable to perform screening tests in populations with a high prevalence or increased risk of the disease [29, 30]. For this reason, the Nurse-WIS was complemented by an entry criterion so that the scale is mainly used for nurses who exhibit the first signs of musculoskeletal disease but have not yet sought medical help. In addition, a cohort of nurses aged 40+ was selected, as long-term sick leave occurs more frequently with increasing age [15].

In the first study on the German version of the Nurse-WIS, information about sick leave was provided by the nurses themselves [27, 28]. In cooperation with DAK-Gesundheit the scale has now been applied in a prospective study in nurses. Because of this cooperation, it was possible to use the health insurance fund’s secondary data on sick leave with the corresponding diagnoses. Therefore an additional objective of the study was to investigate the predictive value of the Nurse-WIS for the duration of sick leave. The hypothesis is that nurses with high risk according to the Nurse-WIS at baseline take longer periods of sick leave due to musculoskeletal diseases and/or psychological impairments than other nurses. This aspect is interesting as the probability of early retirement increases with longer periods of sick leave [31, 32]. Moreover, the model was used to explore whether the duration of sick leave is influenced by other factors such as age, gender, or frequently long and irregular working hours or rotating shifts.

## Methods

### Study design

In cooperation with DAK-Gesundheit, a prospective cohort study was performed with employed nurses aged 40 years and above. The study combined questionnaires and secondary insurance data.

### Setting

The cohort was examined at two different points in time. Baseline measurements were performed in autumn 2011, when the nurses completed a standardised questionnaire.

The DAK-Gesundheit prepared a pseudonymised secondary dataset for the follow-up one year later (2012) and also reported which subjects were still unable to work on 31 December 2012. Continuations of sick-leaves into 2013 cannot be ruled out.

### Participants and data protection

In order to fulfill all the guidelines on data protection, the process of recruitment of the cohort was done as specified by Scharnetzky et al. 2013 [33] as described here.

#### Definition of the cohort

A total of 4500 nurses were selected randomly from the DAK-Gesundheit database of insured persons, based on the following criteria:

- The person was a certified and registered nurse or nursing assistant (DEÜV Social Insurance Code *Occupation* Key 853 and 854).
- The person was professionally active, i.e. not unemployed or unwaged.
- The person was aged ≥40 years and ≤ 65 years.

In addition to these inclusion criteria, the following exclusion criteria were defined:

- Now working in another professional field (e.g. secretary).
- Receiving a pension for reduced work capacity before the survey period.
- Looking for work or taking parental leave.
- Leaving the DAK or death during the survey period.

#### Baseline

In order to guarantee data protection, the data of the defined cohort of nurses was pseudonymised. For this purpose, DAK-Gesundheit randomly generated an identification number (ID) for each nurse of the cohort. This ID was printed on the declaration of consent and on the questionnaire. At Baseline these and other study documents (participant information, data protection information, prepaid return envelope) were posted by the DAK-Gesundheit to the defined cohort. The participating nurses then returned the signed declaration of consent and the completed questionnaire to an independent and confidential study centre (University Medical Centre Hamburg-Eppendorf, German Centre for Health Services Research in Dermatology) in the prepaid envelope. The declaration of consent (with the name and signature of the study participant) was separated from the questionnaire there and sent to the DAK-Gesundheit. The completed questionnaires were sent to the researcher at the CVcare (Competence Centre for Epidemiology and Health Services Research for Healthcare Professionals, University Medical Center Hamburg-Eppendorf).

#### Follow-up

For the follow-up, CVcare compiled a list with the IDs printed on the questionnaires and transmitted this list to DAK-Gesundheit. For this defined group, DAK-Gesundheit prepared a pseudonymised dataset with the secondary data, replacing name, address and other identification characteristics by the corresponding ID. A non-responder analysis was not possible, as all data relating to not participating nurses were deleted for data protection reasons. The dataset was then combined with the baseline data based on the ID by the CVcare researchers. In this way the researchers had no access to the declaration of consent with the name or signature of the study participant and DAK-Gesundheit had no access to the completed questionnaires. For analysis, the data from the baseline questionnaire was linked to the secondary data from the follow-up. In December 2013, DAK-Gesundheit provided administrative data records to the researcher.

This procedure was checked and approved by the Hamburg Commissioner for Data Protection and Freedom of Information. In addition, the Ethics Committee of the Hamburg Medical Association approved the study (No. PV3869).

### Variables and data sources

#### Exposure

As mentioned above, the hypothesis is that nurses with a high risk score at baseline of the Nurse-WIS take longer periods of sick leave due to musculoskeletal diseases and/or psychological impairments. The German version of the Nurse-WIS is based on the original English questionnaire of Gilworth et al. [23] and its underlying concept of work instability as described by Harling et al. [27, 28]. In the present study, an inclusion criterion was added to the German version of the Nurse-WIS in order to identify nurses who had experienced recent signs of a musculoskeletal disease. Only subjects who reported significant musculoskeletal symptoms (lasting more than two hours at a time) in the previous three months were asked to complete the Nurse-WIS. For those who did not fulfill this criterion, the score of the Nurse-WIS was considered as zero. The German version of the scale consists of 28 items covering different aspects of work instability, such as musculoskeletal complaints caused by certain tasks and different psychosocial factors. The individual questions in the scale can be answered with “agree” (=1) or “disagree” (=0) [23, 27]. To calculate the cumulative scores, the points are added up. The greater the value of this cumulative score, the greater the risk of work instability. The score ranges from zero to 28 and is grouped into four categories. With 0 points, there is no risk, or the scale was not applied because of the initial question. With 1 to 9 points, there is a slight risk, with 10 to 19 points a moderate risk, and with 20 to 28 points a high risk is assumed.

#### Outcome variable

For the outcome variables, secondary insurance data of the DAK-Gesundheit on sick leave was used. This dataset included at least one ICD Code (International Statistical Classification of Diseases and Related Health Problems) for each period of sick leave. As previously stated, the secondary dataset included data for the follow-up year (2012) and also reported which subjects were still unable to work on 31 December 2012.

In the data record of the health insurance fund, you can only differentiate between main and secondary diagnoses for inpatient diagnoses, whereby the main diagnosis was used to create the outcome variable. For data from the outpatient sector, it is possible that several diagnoses are specified on sick leave, but no distinction is made between main and secondary diagnoses. For this reason, all diagnoses for the formation of the outcome variable were selected for the outpatient data as soon as a musculoskeletal or mental illness was present.

As described in the literature [36–38] diseases of the back and the upper extremities are of particular interest as occupational risk factors. Moreover disorders in psychological wellbeing, stress disorders and impairments such as depression and burnout are often associated with acute and chronic musculoskeletal diseases [39–41].

Based on the ICD Code and the corresponding number of days of absence in each individual period of sick leave, diverse outcome variables for sick leave due to a musculoskeletal (ICD Code M40-M54) and/or psychological impairment (ICD Code F32-F48, Z73) were defined as explained below.

#### Potential confounders

In addition to the Nurse-WIS, the standardised questionnaire at baseline contained questions concerning sociodemographic characteristics (e.g. gender, age, occupational training) and the occupational situation (e.g. length of service in that occupation) as well as questions concerning sick leave over the previous 12 months before baseline (2011). Accordingly, the secondary data set also includes data on occupation [DEÜV-Social Insurance Code], date of birth and gender.

This information was used to construct the following variables, which were considered as potential confounders:

- age,
- gender,
- type of work (administrative, nursing, equal amounts of administrative and nursing work),
- shift (day duty, always at the same time, rotating shift excluding nights, rotating shift including nights, only night work),
- facility (clinic or hospital, old people’s home, facility for the handicapped),
- training (diploma in nursing, nursing assistant, without training),
- absenteeism due to musculoskeletal disease and/or psychological impairment in the previous year before baseline (2011).

### Statistical analysis

#### Assessment of the predictive characteristics of the nurse-WIS for long-term sick leave

The following parameters were used to assess the performance of the Nurse-WIS in relation to long-term sick leave during the follow-up period, i.e. 2012: sensitivity, specificity, PPV, NPP and likelihood ratios. Long-term sick leave was defined as a sick leave of more than six weeks (> 42 days) due to musculoskeletal diseases and/or psychological impairments in 2012.

#### Prognostic influence of the nurse-WIS on the duration of sick leave

As mentioned above, the hypothesis is that nurses with high risk according to the Nurse-WIS at baseline take longer periods of sick leave due to musculoskeletal diseases and/or psychological impairments. In order to consider the association between the Nurse-WIS and sick leave in the following year (2012) in a more differentiated manner, a multiple ordinal logistical model (proportional odds model) was used. For this purpose, the duration of sick leave was divided into the following categories:

- no sick leave (0 days),
- sick leave up to 6 weeks (1–42 days),
- sick leave from 6 weeks to 12 months (43 days to 364 days),
- sick leave of 12 months or more (≥365 days, still unable to work after 31 December 2012).

Aside from the Nurse-WIS, potential confounders as described above were considered. Based on these factors, step-wise backward selection using likelihood ratio tests at a significance level of 0.05 was performed. After each step, it was examined whether the respective factor was a confounder for the Nurse-WIS (defined as a change of ≥10% in the coefficients of the Nurse-WIS relative to the full model). Factors identified as confounders remained in the model. We checked the proportional odds assumption of the ordinal logistic model by performing a likelihood ratio test (significance level 0.05) between the ordinal and the corresponding multinomial model.

For the final model, odds ratios (OR) with corresponding 95% confidence intervals (95%CI) and Wald *p* values are reported. In addition, for the Nurse-WIS the predicted marginal probabilities (as means over participants) are given with corresponding 95% confidence intervals (95%CI). The analyses were conducted with Stata 14.1 (StataCorp 2015, College Station, TX).

## Results

### Study population

**Fig. 1:**
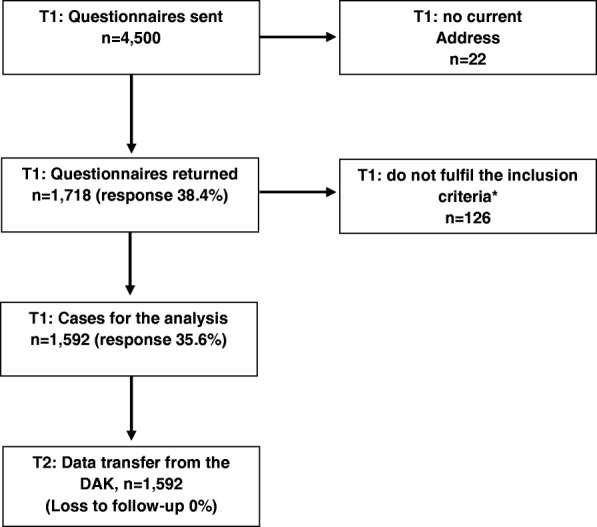
Flow chart of the study population at baseline (T1) and after follow-up (T2)

A total of 1592 nurses took part in the baseline survey (response rate 35.6%). By using the ID at follow-up, the data of all the participants in the baseline survey could be linked to the sick leave data of the health insurance fund (loss to follow-up 0%) (Fig. 1). The characteristics of the study population are shown in Table 1. Most of the nurses were female (91.8%). About 30% of the study participants were 50–54 years old, 88.4% had received nursing training, 84.5% worked in a hospital and 55.5% had worked in nursing for more than 30 years. Most of the study participants were in full-time employment (55.0%) and either worked in rotating shifts excluding nights (30.6%) or including nights (45.2%). Most study participants (57.3%) performed equal amounts of nursing and administrative work. While 36.4% reported that they mainly performed nursing work, only 6.3% reported that they mainly performed administrative work. According to the Nurse-WIS, 19.7% have no risk, 7.7% a slight risk, 33.2% a moderate risk and 39.4% a high risk of a long-term sick leave (Table 1).

**Table 1:** Description of the study population and the categories of the Nurse-WIS (*n* = 1592)

Variables	% (n)
Gender	
Female	91.8% (1462)
Male	8.2% (130)
Age	
40 to 44 years	18.0% (286)
45 to 49 years	26.1% (416)
50 to 54 years	29.0% (462)
55 to 59 years	21.0% (334)
> 60 years	5.9% (94)
Occupational training	
Qualified nurse or geriatric care worker	88.4% (1408)
Nursing assistant without nursing training	11.6% (184)
Facility	
Clinic or hospital	84.5% (1346)
Old people’s home, nursing home, facility for the handicapped	15.5% (246)
Length of service	
0–20 years	9.7% (154)
21–30 years	34.9% (555)
More than 30 years	55.5% (883)
Scope of employment	
Full-time	55.0% (875)
Part-time (< 35 h a week)	45.0% (717)
Working hours	
Rotating shifts excluding nights	30.6% (487)
Rotating shifts including nights	45.2% (720)
Day duty, always at the same times	17.0% (271)
Only night work	7.2% (114)
Principal activity	
Nursing work	36.4% (579)
Administrative work	6.3% (100)
Equal parts of both	57.3% (913)
Nurse-WIS	
No risk (0 points)	19.7% (313)
Slight risk (1–9 points)	7.7% (123)
Moderate risk (10–19 points)	33.2% (528)
High risk (20–28 points)	39.4% (628)

### Sick leave during the follow-up

Sick leaves of at least one day were most often due to other diseases. While 27.4% of sick leave periods were due to musculoskeletal diseases and 16.3% to psychological impairment. 40.1% of participants took sick leave of at least one day due to musculoskeletal diseases and/or psychological impairments. As regards the duration of sick leave, the proportion of persons who took less than 6 weeks’ sick leave was greatest in the diagnostic group of other diseases. Sick leave of > 6 to 12 weeks was also most frequent in other diseases (10.1%). However, sick leave of more than 12 months was more frequently due to a musculoskeletal disease or a psychological impairment (Table 2).

**Table 2:** Sick leave in the follow-up depending on the disease

	For other diseases	Due to a musculo-skeletal disease	Due to a psycho-logical impairment	Due to musculoskeletal disease and/or psychological impairment
Sick leave	% (n)	% (n)	% (n)	% (n)
No	54.1% (861)	72.6% (1155)	83.7% (1333)	59.9% (953)
Yes (at least 1 day)	45.9% (731)	27.4% (437)	16.3% (259)	40.1% (639)
Length of sick leave				
Up to 6 weeks (1–42 days)	34.9% (556)	21.8% (347)	11.6% (185)	29.6% (472)
> 6 weeks – 12 months (43–364 days)	10.1% (161)	4.1% (65)	3.4% (54)	7.6% (121)
> 12 months (**≥**365 days)	0.9% (14)	1.6% (25)	1.3% (20)	2.9% (46)

### Association between the nurse-WIS and the duration of sick leave

**Fig. 2:**
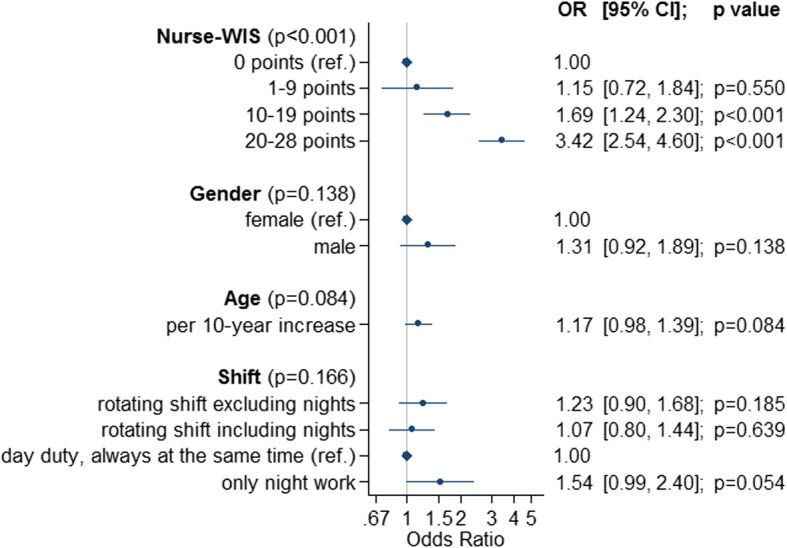
Odds Ratio for sick leave due to musculoskeletal disease and /or psychological impairment depending on Nurse-WIS, Gender, Age and Shift Work

Of the potential prognostic factors examined, only the Nurse-WIS had a significant influence on the duration of sick leave (*p* < 0.001). The factors age, gender and shift work were identified as confounders of the Nurse-WIS and were therefore considered in the final regression model. The proportional odds ratio for a Nurse-WIS point value between 10 and 19 (moderate risk) relative to 0 points (no risk) for the duration of sick leave was 1.69 (95% CI [1.24, 2.30]; p < 0.001) (Fig. 2). In more detail, this means that the odds of taking sick leave of more than 1 day (i.e. for the combined categories of up to 6 weeks, 6 weeks to 12 months and more than 12 months) are 1.69 times higher than for nurses with a zero score in the Nurse-WIS. Because of the proportional odds assumption, the odds ratios for sick leave of more than 6 weeks versus less than 6 weeks, as well as (more than) one year versus less than one year, are also 1.69. The proportional odds ratio relative to no risk (0 points) increases for higher point values in the Nurse-WIS. At slight risk (1–9 points), the odds ratio in comparison to no risk was not increased (1.15 (95%CI [0.72, 1.84]; *p* = 0.550)). However, at high risk (20–28 points), the odds ratio increased to 3.42 (95%CI [2.54, 4.60] *p* < 0.001).

**Fig. 3:**
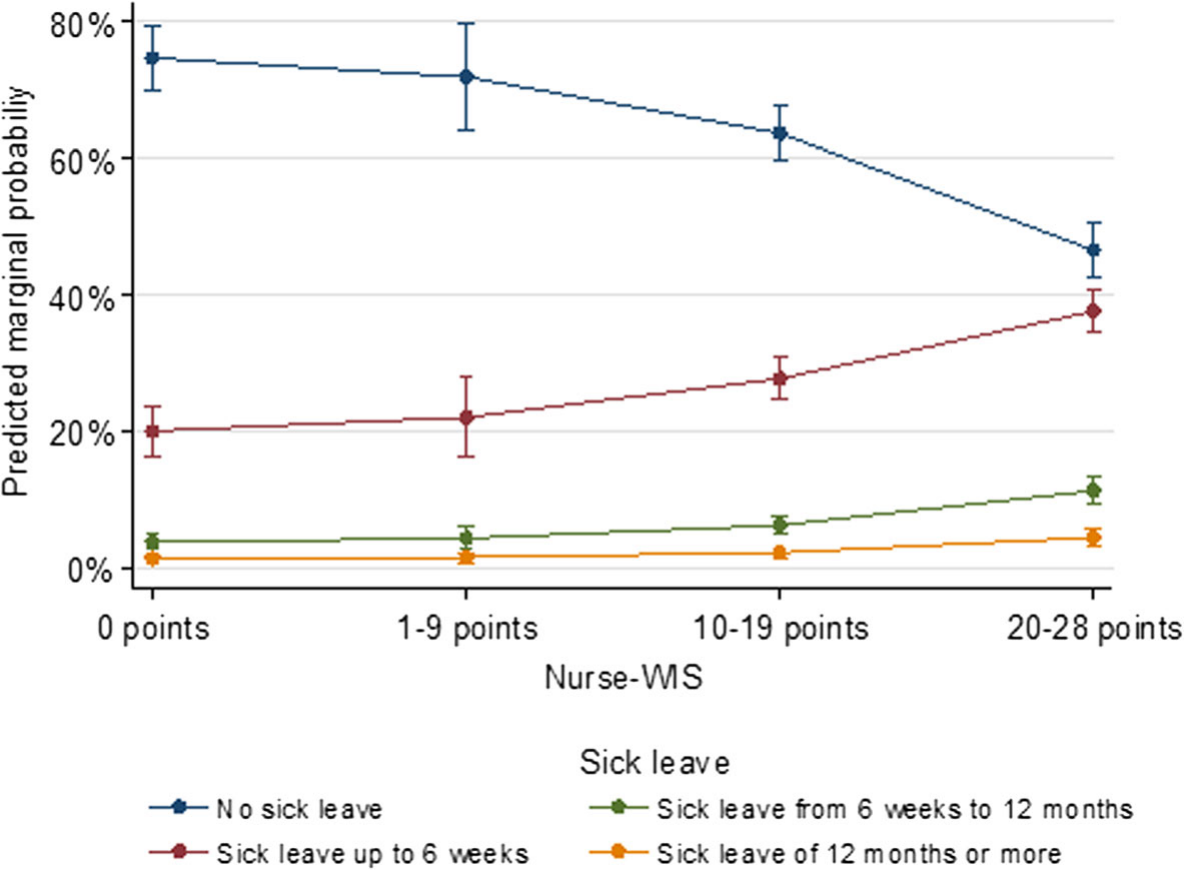
Adjusted predicted marginal probabilities for duration of sick leave due to a musculoskeletal disease and/or psychological impairment by Nurse-WIS risk category

This relationship is also reflected in the predicted marginal probabilities (Fig. 3). With increasing risk according to the Nurse-WIS, the predicted probability for longer sick leave due to a musculoskeletal disease and/or a psychological impairment increases. With no, slight or moderate risk according to the Nurse-WIS, the predicted probabilities for no sick leave are 74.7% (96%CI [70.0%, 79.5%]), 71.9% ([64.0%, 79.9%]) and 63.7% ([59.7%, 67.7%]) respectively, while it was only 46.6% ([42.7%, 50.4%]) for 20 points and more (high risk). In contrast, the predicted probability for sick leave of up to 6 weeks was 20.1% for a Nurse-WIS of 0 points, 22.1% for 1–9 points, 27.8% for 10–19 points and 37.6% for 20–28 points. For a high risk of 20–28 points, the predicted probability of long-term sick leave of 6 weeks to 12 months was 11.4%, compared to 3.9% for no risk.

In the high risk group (Nurse-WIS 20–28 points) the sensitivity for long-term sick leave during the follow-up was 64.1% and the specificity was 63.4. The PPV was 17.0 and the NPV 93.8% (Table 3).

**Table 3:** Sensitivity, specificity, likelihood ratio and predictive value of the Nurse-WIS for long-term sick leave^1^ during follow-up

	% (n)
High risk according to Nurse-WIS	39.4 (628)
Long-term sick leave^1^	10.9 (173)
Sensitivity	64.1
Positive likelihood ratio^2^	1.75
Specificity	63.4
Negative likelihood ratio^2^	0.57
Positive predictive value (PPV)	17.0
Negative predictive value (NPV)	93.8

^1^Long-term sick leave > 42 days due to musculoskeletal diseases and/or psychological impairment (e.g. burn-out)

^2^No unit

## Discussion

To our knowledge, this is the first study to have analysed the prevalence of work instability in nurses and the predictive value of a high score with regard to long-term sick leave during follow-up by combining survey data with secondary data of a health insurance fund. The prevalence of a high risk of work instability was quite high (39.4%). One nurse out of ten took long-term sick leave due to musculoskeletal diseases and/or psychological impairment during follow-up. Even though the odds ratio for sick leave increased with a Nurse-WIS > 10 points, the sensitivity and specificity of the Nurse-WIS with regard to long-term sick leave during follow-up were rather modest, which is also reflected in a low PPV (17Compared with our previous study of geriatric care workers, sensitivity and specificity of the Nurse-WIS was lower (sensitivity 73.9 versus 64.1 specificity 76.7 versus 63.4) [27]. Therefore the attempt to improve predictive values by introducing an additional variable, pain during the previous three months, was not successful.

As the predictive value depends on the prevalence of the disease, only persons aged 40+ were surveyed, as the risk of sick leave increases with age [15]. In addition, an entry question was added to the questionnaire, so that only those persons who had suffered symptoms lasting longer than 2 h in their musculoskeletal system over the previous 3 months were requested to complete the questionnaire. The predictive characteristics of the updated version of the scale with the entry criterion were then compared with those of the geriatric care workers study. In the geriatric care workers study, 28.4% of the subjects reported a high risk in the Nurse-WIS. This was lower than the corresponding value for nurses (39.4%). This may be explained by the higher age of the cohort of nurses, as it has been shown that the probability of increased risk in the Nurse-WIS increases with age [27]. What was astonishing was that the proportion who had taken long-term sick leave due to a musculoskeletal disease and/or a psychological impairment was similar (about 10%) in both studies. A higher value had been expected in the cohort of nurses which was restricted to older nurses. The two studies used the same definition of long-term sick leave, although the underlying data was quite different. In the geriatric care workers study, periods of sick leave were calculated based on information provided by the subject. They may be distorted by recall bias, particularly when the sick leave was some time earlier. In the nurse study this data was taken from the health insurance fund and had therefore been systematically collected and electronically recorded in the context of administration or cost reimbursement [42]. We assume that it is therefore more valid. Furthermore, different follow-up rates might be responsible for divergent results in the two studies.

### Association between the nurse-WIS and the length of sick leave

A second objective of the study was to determine the prognostic influence of the Nurse-WIS in a multivariable model on the duration of sick leave (no sick leave, up to 6 weeks, > 6 weeks to 12 months, ≥12 months). Here, only the Nurse-WIS had a significant influence on the duration of sick leave during follow-up in 2012, with proportional odds of 3.42 for a Nurse-WIS score 20 to 28. This supports the hypothesis that nurses with an increased score in the Nurse-WIS have a higher risk of a longer sick leave [31, 32]. As the probability of early retirement increases with the length of a sick leave, the Nurse-WIS appears to be suited to detect persons at risk. For this reason, it is probable – even though the predictive values of the scale were poorer than in the geriatric care workers study [28] – that the Nurse-WIS can provide at least initial evidence that a person is at risk of a long sick leave and might benefit from intervention. It would also be conceivable to have the result of the Nurse-WIS confirmed by additional investigation by an occupational therapist, company doctor or other physician.

The factors age, gender, and shift work were identified as confounders. This means that age influences both the length of sick leave and the Nurse-WIS. This is well in line with the observation that the general state of health and length of sick leave change with age in several occupational groups, including nurses [34, 35]. It has also been suggested that frequently long and irregular working hours and rotating shifts may influence the development of disease [43, 44]. These factors could be considered when the Nurse-WIS is used in future, for example by inclusion in the questionnaire.

### Cooperation with the health insurance fund and special features of the study design

The present study was performed in cooperation with DAK-Gesundheit, which has about 4.9 million members and 6 million insured persons. As the DAK was originally a health insurance fund for employees, it now typically insures employees in jobs typically done by women (e.g. in the health service, retail, office work and administration). The health service is an economic sector that employs particularly large numbers of DAK members [45]. Thus DAK-Gesundheit was a suitable partner in the study on validation of the Nurse-WIS. Moreover, this cooperation permitted the use of secondary data. One special feature of the study was the design. As in Scharnetzky et al. 2013 [33], this employed a procedure comprising interviews of insured persons followed by linkage of this data with the secondary health insurance fund’s data for this cohort (with their consent). There was no loss of follow-up, which is a considerable advantage of this approach. Loss of follow-up might be an explanation of the divergent results of the nurses and geriatric care workers study [27]. It can be assumed that the sick leave data from the health insurance fund is more valid than the information obtained by means of a questionnaire. In addition, sick leave always corresponds to a restriction in working capacity as certified by a doctor and is not equivalent to the presence of a disease. It must therefore be assumed, that the sick leave data contains both underestimates (“presentism”) and overestimates [46]. Furthermore, sick leave assessment on the basis of administrative data omits some short-term sick leave. In Germany, sick leave of up to three days does not usually require certification by a physician. Therefore, short-term sick leave was underestimated. This might have introduced some non- differential misclassification most likely diluting the effect estimates.

Nevertheless, it has been shown that data from the social insurance system is suitable for scientific analysis. For example, a Danish study used secondary data to show length of sick leave to be a predictor for receiving disability pension in future [31].

### Limitations

Our study has some limitations. The response rate of 35.6% is low and therefore the results are not necessarily transferable to the total group of nurses. Furthermore, a selection bias cannot be excluded and may have led to an overestimation or underestimation. Because of the data collection by the DAK, as described in the method section, no non- responder analysis could be performed and we unfortunately have no information about the group of non-responders.

In this study an additional variable (pain during the last three months) was used to improve the predictive value compared to previous studies. Unfortunately, this attempt has not been successful. Due to the low positive predictive value, the question arises whether the Nurse-WIS is well suited as a screening tool. Even if the predictive values of the scale have deteriorated compared to the elderly care study, it is likely that the Nurse WIS can provide at least a first indication of persons who are at risk of a longer sick leave and who would possibly benefit from early intervention measures. However, it should be discussed whether the result of the nurse WIS should be confirmed by an additional examination by an occupational therapist, company physician or physician. The factors age, gender, and shift work were identified as confounders. However, we do not have information about occupational risk factors or live style factors so that controlling for confounding is limited in our analysis.

## Conclusion

The German version of the Nurse-WIS predicts long-term sick leave. Introducing an entry criterion did not improve the predictive value of the score. Restricting the cohort to nurses aged 40 and over increased the proportion of those with a high risk score but did not improve prediction of long-term sick leave compared with the previous geriatric care worker study. Nevertheless, our data corroborates the hypothesis that the Nurse-WIS is a useful tool for assigning rehabilitation programmes to nurses. Combining questionnaire data with secondary data from a health insurance fund was feasible and successful in terms of follow-up. Therefore further studies employing this combination of data are recommended.

## Abbreviations

DAK: Health Insurance Fund Board Manager for Health Care Research
DEÜV: Social Insurance Code Occupation Key
ICD Code: International Statistical Classification of Diseases and Related Health Problems
Nurse-WIS: Nurse-Work Instability Scale
OR: Odds Ratio
PPV: Positive predictive value
LR +: Positive likelihood ratio
LR-: negative likelihood ratio
